# A Proposal to Stratify the Intermediate-Risk Thyroid Nodules According to the AACE/ACE/AME Guidelines with Ultrasound Features

**DOI:** 10.1038/s41598-017-18207-y

**Published:** 2017-12-20

**Authors:** Xiao-Hong Deng, Li-Na Tang, Shui-Qing Liu, Xiao-Long Li, Ya-Ping He, Hui-Xiong Xu

**Affiliations:** 1 Department of Medical Ultrasound, Shanghai Tenth People’s Hospital, Ultrasound Research and Education Institute, Tongji University School of Medicine, Shanghai, 200072 China; 2 Department of Medical Ultrasound, Fujian Cancer Hospital & Fujian Medical University Cancer Hospital, Fuzhou, 350014 China; 3 Department of Medical Ultrasound, Changzhou First People’s Hospital & The Third Affiliated Hospital of Suzhou University, Changzhou, 213003 China; 4 Thyroid Institute, Tongji University School of Medicine, Shanghai, 200072 China; 5 Shanghai Center for Thyroid Diseases, Shanghai, 200072 China

## Abstract

To propose a risk stratification system for intermediate-risk thyroid nodules (TNs) according to American Association of Clinical Endocrinologists, American College of Endocrinology and Associazione Medici Endocrinologi Medical (AACE/ACE/AME) Guideline with ultrasound (US) features. 1000 patients with 1000 nodules (902 benign nodules and 98 malignant nodules) were included. All the nodules were confirmed with either fine needle aspiration (FNA) cytology and follow-up or histology results after surgery. Univariate analysis and binary multivariate logic regression analysis were applied to analyze the possible risk US features associated with malignancy. Receiver operating characteristic curves (ROC) were drew and compared. Univariate analysis and binary multivariate logistic regression analysis showed that indeterminate hyper-echoic spot (*OR* = 4.544), slightly ill-defined margin (*OR* = 2.559), slight hyper-echogenicity (*OR* = 1.992) and no macro-calcification (*OR* = 1.921) were risk factors for the intermediate-risk thyroid nodules (TNs). A predicting model was established based on the 4 risk factors. The risk rates of malignancy were 5.7% (26/455) in Stage I, 11.0% (49/445) in Stage II, 23.1% (21/91) in Stage III, 33.3% (3/9) in Stage IV. In conclusion, for the intermediate-risk TNs, special attention should be paid to the TNs with indeterminate hyper-echoic spot, slightly ill-defined margin, slight hyper-echogenicity, or no macro-calcification. The probability of malignancy increased with the number of risk factors increasing.

## Introduction

With wide application of high resolution ultrasound (US) in clinic practice, more and more thyroid nodules (TNs) are discovered. Over 50% of healthy people are detected with TNs^1^ and 7–15% of them are malignant^2^. The malignancy rate of all thyroidectomies has increased to 41.5% in recent years^3^. Most of the TNs are clinically asymptomatic. Therefore, the main challenge in the management of TNs is to identify malignancy, with US and fine-needle aspiration (FNA) biopsy as the main diagnostic cornerstones. American Association of Clinical Endocrinologists (AACE), American College of Endocrinology (ACE) and Associazione MediciEndocrinologi (AME) Medical Guidelines are practical clinic statements for thyroid diagnosis and management. The first edition was published in 2006, while the latest edition was released in 2016^1^.

According to the guidelines, the following US thyroid rating system for TNs is suggested. Type 1. Low-risk nodules, including: (a) mostly cystic (>50%) nodules with reverberating artifacts without suspicious US signs; (b) isoechoic confluent spongiform nodules, or isoechoic nodules with regular halo. The expected malignancy risk of type 1 is about 1%. Type 2. Intermediate-risk nodules: (a) isoechoic and slightly hypoechoic nodules, with ovoid-to-round shape and well or slightly ill-defined margins; (b) these nodules may have macro or continuous rim calcifications, intra-nodular blood flow or hyperechoic spots of uncertain significance. The expected malignancy risk is 5–15%. Type 3. High-risk nodules with at least one of the following suspicious features: (a) remarkable hypo-echogenicity; (b) micro-lobulated or spiculated margins; (c) micro-calcifications; (d) taller-than-wide shape; (e) evidence of extra-thyroidal growth. The expected malignancy risk is 50–90%.

The recommended management for low-risk TNs is follow-up while for high-risk TNs is FNA or surgery. However, for intermediate-risk TNs, the management is still controversial. As 5–15% of intermediate-risk nodules are malignant, there is still a need to stratify those nodules and rule out the malignancy to reduce unnecessary FNA or surgery. According to the AACE/ACE/AME guidelines, FNA is only recommended for the intermediate US risk TNs >20 mm. Unfortunately, few relevant data were available for those intermediate-risk TNs in the literatures. In the current study, it was aimed to find out the suspicious US features among the intermediate-risk TNs, which would stratify those nodules and facilitate subsequent management.

## Results

### Patients and nodules

**Figure 1:**
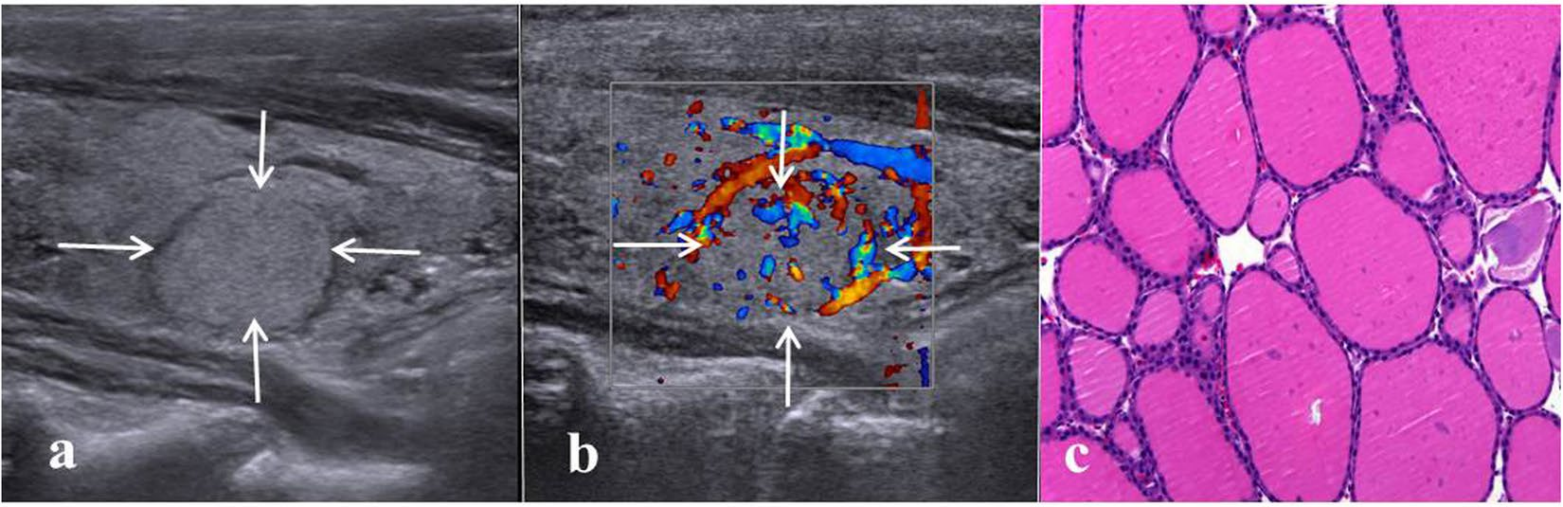
Images in a 61-year-old woman with a 13-mm nodular goiter in the right thyroid lobe. (**a**) Conventional US shows the features of iso-echogenicity and well defined margin. (**b**) Color Doppler US shows predominately peri-nodular blood flow. (**c**) Histologic examination (haematoxylin-eosin staining, ×200) confirms the diagnosis of nodular goiter.

**Figure 2:**
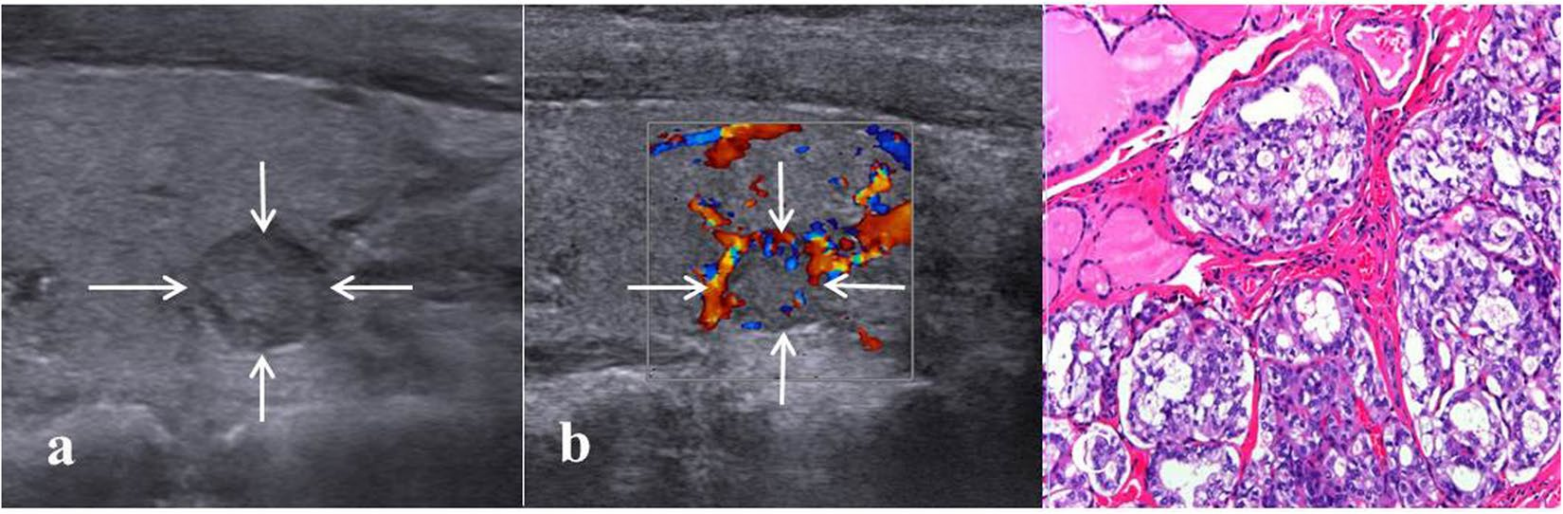
Images in a 31-year-old woman with an 8-mm papillary thyroid carcinoma in the left thyroid lobe. (**a**) Conventional US shows features of slight hypo-echogenicity and no macro-cacification. (**b**) Color Doppler US shows predominately peri-nodular blood flow. (**c**) Histologic examination (haematoxylin-eosin staining, ×200) confirms the diagnosis of papillary thyroid carcinoma.

**Figure 3:**
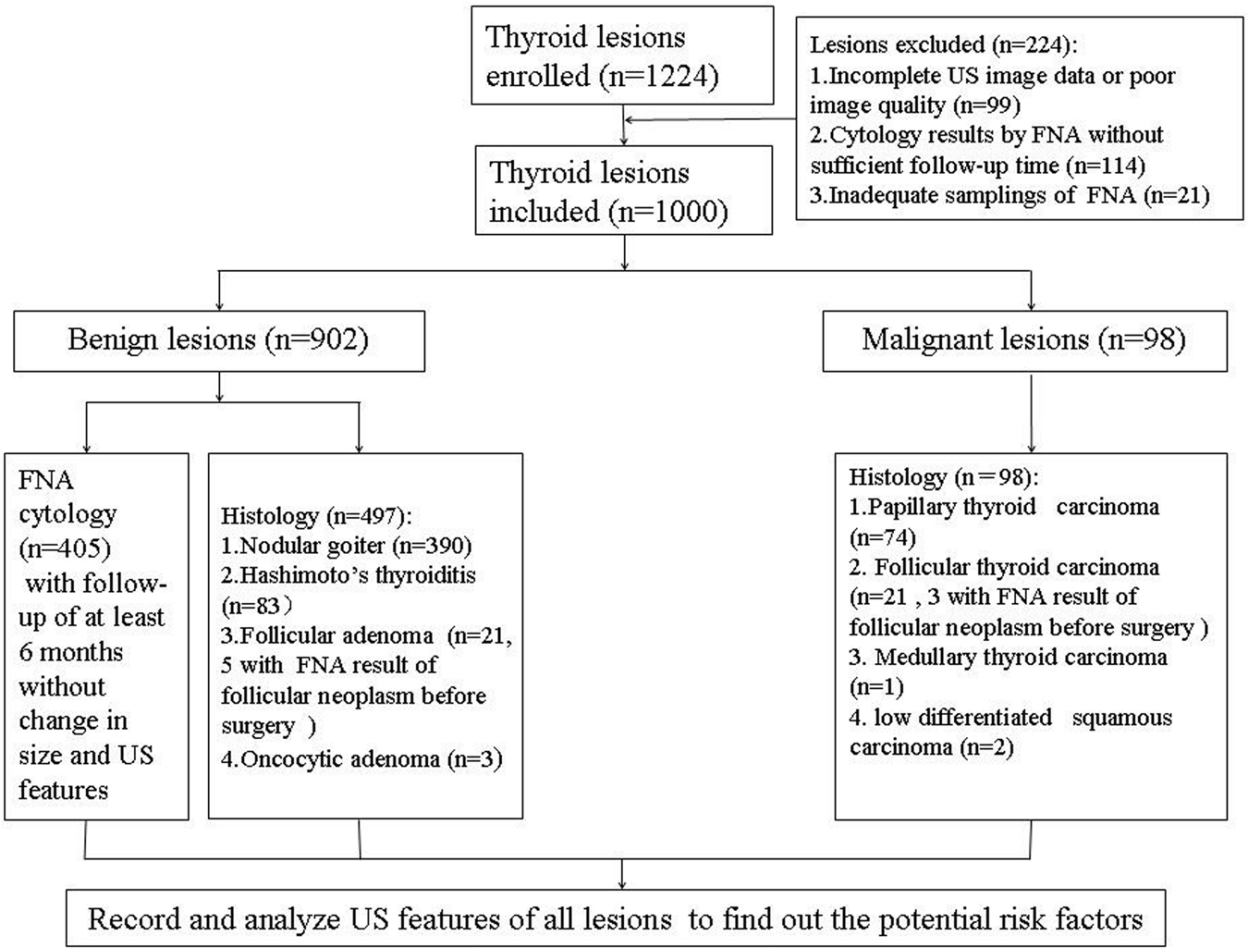
The flowchart of nodule selection and design of the study.

The malignancy rates for all the 1000 nodules, those >20 mm and those ≤20 mm group were 9.8% (98/1000), 7.5% (29/374), 11% (69/626) respectively. For the benign nodules, 405 were proved by FNA cytology with at least 6 months’ follow-up while 497 were proved by histology after surgery. For the 497 benign nodules proved by histology after surgery, the diagnoses were nodular goiter (n = 390) (Fig. 1), Hashimoto’s thyroiditis (n = 83), follicular adenoma (n = 21), oncocytic adenoma (n = 3). Among the 98 malignant nodules proved by histology after surgery, the diagnoses were papillary thyroid carcinoma (n = 74) (Fig. 2), follicular thyroid carcinoma (n = 21), medullary thyroid carcinoma (n = 1), and low differentiated squamous carcinoma (n = 2). 8 TNs revealed follicular neoplasms by FNA cytology, and then all underwent surgery after FNA, so they were included in histology groups(5 follicular adenoma and 3 follicular thyroid carcinoma). The flowchart of nodule selection and the design of the study are shown in Fig. 3.

### Univariate analysis

In univariate analysis, younger patient age, smaller nodule maximum diameter, slight hypo-echogenicity, slightly ill-defined margin, no macro-calcification and indeterminate hyper-echoic spot were significantly associated with malignancy (all *P*  < 0.05) (Table 1). Benign nodule was significantly larger than malignant one, however, there was no statistical difference between those ≤20 mm and those >20 mm. Conversely, patient gender, nodule location, internal component, echo uniformity and vascularity did not achieve significant differences (all *P* > 0.05) (Table 1).

**Table 1:** Comparisons on US features between the benign group and the malignant group.

*Characteristics*	*Benign group (n* = *902)*	*Malignant group (n* = *98)*	*P*
*Gender*			0.750
*Female*	703(77.9)	75(76.5)	
*Male*	199(22.1)	23(23.5)	
*Age* ^#1^	52.8 ± 12.8	49.0 ± 14.0	0.007*
*Maximum diameter* ^#2^	16(18)	10.5(21.25)	<0.001*
*Maximum diameter*			0.093
*≤20* *mm*	557 (61.8)	69(70.4)	
>*20* *mm*	345 (38.2)	29(29.6)	
*Margin*			<0.001*
*Well defined*	836(92.7)	79(80.6)	
*Ill-defined*	66(7.3)	19(19.4)	
*Echogenicity*			0.006*
*Iso-echoic*	254(28.2)	15(15.3)	
*Slightly hypo-echoic*	648(71.8)	83(84.7)	
*Location*			0.412
*Left*	406(45.0)	51(52.1)	
*Right*	484(53.6)	46(46.9)	
*Isthmus*	12(1.4)	1(1.0)	
*Component*			0.894
*Solid*	180(20.0)	19(19.4)	
*Predominantly solid*	722(80.0)	79(80.6)	
*Echo uniformity*			0.721
*Uniform*	315(34.9)	36(36.7)	
*Non-uniform*	587(65.1)	62(63.3)	
*Macro-calcification*			0.022*
*Present*	244(27.1)	16(16.3)	
*Absent*	658(72.9)	82(83.7)	
*Vascularity*			0.075
*Type I*	330(36.6)	37(37.8)	
*Type II*	246(27.3)	17(17.3)	
*Type III*	326(36.1)	44(44.9)	
*Indeterminate hyper-echoic spot*			0.010*
*Present*	13(1.4)	5(5.1)	
*Absent*	889(98.6)	93(94.9)	

Numbers in parentheses were percentages or inter-quartile range.

^#1^Age was expressed as mean ± SD, ^#2^maximum diameter was expressed as median (inter-quartile range), while other characteristics were expressed as number(percentage)

*Statistically significant difference.

### Binary multivariate logistic regression analysis

Pathological diagnosis was the dependent variable in binary multivariate logistic regression analysis, and statistically different indexes in univariate analysis (patient age, nodule diameter, echogenicity, margin, macro-calcification and indeterminate hyper-echoic spot) were independent variables. Binary multivariate logistic regression analysis was conducted with *OR* < 1 as protective factors and *OR* > 1 as risk predictor factors. Multivariate logistic regression analysis showed that indeterminate hyper-echoic spot (*OR*: 4.544; 95% CIs: 1.537–13.438) was the most significant independent risk predictor for the intermediate-risk TNs according to ACEE/ACE/AME guidelines, followed by slightly ill-defined margin (*OR*: 2.559; 95% CIs: 1.417–4.620), slight hyper-echo (*OR*: 1.992; 95% CIs: 1.099–3.612) and no macro-calcification (*OR*: 1.921; 95% CIs: 1.085–3.402) (Table 2). Older patient age was discovered to be a protective factor (*OR*: 0.982; 95% CIs: 0.967–0.998) (Table 2).

**Table 2:** Binary logistic regression analysis on US features of TNs.

*Variables*	*B*	*S*.*E*	*P*	*OR*	*OR 95% C*.*I*.	
					lower	upper
*Older patient age*	−0.018	0.008	0.026*	0.982	0.967	0.998
*Maximum diameter*	−0.009	0.010	0.351	0.991	0.973	1.010
*Slight hypo-echogenicity*	0.689	0.304	0.023*	1.992	1.099	3.612
*Slightly ill-defined margin*	0.940	0.301	0.002*	2.559	1.417	4.620
*No macrocalcification*	0.653	0.292	0.025*	1.921	1.085	3.402
*Indeterminate Hyper-echoic spot*	1.514	0.553	0.006*	4.544	1.537	13.438

US = ultrasound; B = regression coefficient; SE = standard error for regression coefficient; Sig = significance; *OR* = odds ratio.

*Statistically significant difference.

A binary multivariate logistic regression predictive equation was then set up as follows: P = 1/1 + Exp ∑ [−12.683 + 0.689 × (if slight hypo-echogenicity) + 0.940 × (if ill-defined margin) + 0.653 × (if none macro-calcification) + 1.514 × (if indeterminate hyper-echoic spot)]. With the number of risk factors increasing, the probability of malignancy increased. ROC curves were plotted to evaluate the diagnostic performances of the predictive equation, nodule echogenicity, margin, macro-calcification and indeterminate hyper-echoic spot. The areas under the curve (AUCs) were 0.660, 0.564, 0.560, 0.553 and 0.518, respectively. In terms of AUC, the predictive equation achieved the highest diagnostic performance (all *P* < 0.05). The best cut-off value for the predictive equation was −0.13 (YI = 0.276). The sensitivity and specificity were 45.9% and 81.7% respectively. For TNs >20 mm, the AUC, sensitivity and specificity of the equation were 0.566, 58.6%, 57.4% respectively; for TNs ≤ 20 mm, the AUC, sensitivity and specificity were 0.700, 59.4%, 76.5% respectively. The AUC for TNs ≤ 20 mm was higher than that for TNs ≤ 20 mm (*P* < 0.05).

A predicting model was established based on the 4 risk factors from the binary multivariate logistic regression analysis. Then risk score (RS) for each nodule was calculated as follows: RS = 0.7 × (if slight hypo-echogenicity) +0.9 × (if ill-defined margin) +0.7 × (if none macro-calcification) +1.5 × (if indeterminate hyper-echoic spot). The rating system was divided as following: Stage I, RS was <0.7 and none of 4 risk factors was enrolled, including 455 patients (45.5%); Stage II, RS was 0.7 to 1.5 and any 1 of 4 risk factors was enrolled, including 445 patients (44.5%); Stage III, RS was 1.6 to 2.4 and any 2 of 4 risk factors were enrolled, including 91 patients (9.1%); Stage IV, RS was 2.5 to 3.1 and any 3 of 4 risk factors were enrolled, including 9 patients; Stage V, RS was 3.2 to 3.8 and all of 4 risk factors were enrolled, no patient was included.

The risk rates of malignancy were 5.7% (26/455) in Stage I, 11.0% (49/445) in Stage II, 23.1% (21/91) in Stage III, 33.3% (3/9) in Stage IV. With these findings, we regarded Stage I and Stage II (none or 1 risk factor) as low suspicion, and Stage III and Stage IV (2 or 3 risk factors) as mediate suspicion.

## Discussion

In the past few years, several international societies have published different thyroid US risk stratification systems to provide practical guides for thyroidologists^1,2,4^. In particular, AACE/ACE/AME guideline is one of the most popularly adopted guidelines in clinical practice^5^. TNs with high suspicious US features are mostly recommended to FNA or surgery, while nodules with low US risk are often recommended to follow-up. However, how to manage the patients with intermediate risk TNs is uncertain as the malignancy rate is still 5 to 15 percent. The AACE/ACE/AME guideline recommended FNA for the intermediate risk TNs>20 mm. Anyhow, there was no statistically significant difference in malignancy rate between nodules >20 mm and nodules ≤20 mm in the present study, which made the recommendation rule of AACE/ACE/AME guideline questionable for the intermediate risk TNs. Therefore, there is a need to develop a new algorithm to stratify the intermediate risk TNs based on the US features instead of nodule size, the aim of which is to reduce unnecessary FNA or surgery and avoid omission of possible malignancy. Unfortunately, no relevant studies have been reported so far. In the present study, we hypothesized that there were some potential risk US factors of the intermediate risk TNs according to AACE/ACE/AME guidelines.

Indeterminate hyper-echoic spot in the nodule was considered to be the most significant independent risk predictor (*OR*: 4.544; 95% CIs: 1.537–13.438) by binary multivariate logistic regression analysis. Indeterminate hyper-echoic spot is a new concept appeared only in AACE/ACE/AME guidelines and no relevant study has mentioned it before. Homogeneous hyper echogenicity of the nodule was commonly considered to be associated with benign nodules by studies from Xu *et al*.^6^ and Kuru *et al*.^7^. Indeterminate hyper-echoic spot inside the nodule as hybrid ingredient in iso-echoic or slight hypo-echoic TNs, may be caused by reflection of fibrosis or mesenchyme inside the carcerous tissue, further studies are necessary to compare the hyper-echoic spot with pathology components.

Slightly ill-defined margin (*OR*: 2.559; 95%CIs: 1.417–4.620) and slight hypo-echo (*OR*: 1.992; 95%CIs: 1.099–3.612) were also risk factors that cannot be neglected. Kuru *et al*.^7^ found ill-defined margin and hypo-echogenicity were risk factors of malignancy by analyzing 485 TNs. Ill-defined margin was also proved to be a risk factor (*OR* = 3.600) by the study from Batawi *et al*.^8^. Hypo-echogenicity and ill-defined margin, whatever extent they were, were considered as independent risk factors. The aggressive growth of carcinoma may lead to the US feature of ill-defined margin, and fuzzy boundary was prompted by tumor’s infiltrating into surrounding tissue. Carcinoma cells were more than mesenchyme in malignant nodules, thus few US reflection interfaces were created, which may be the underlying mechanisam for hypo-echogenicity.

Nodules without macro-calcification were 1.896 times more dangerous than nodules with macro-calcification in our study (*OR*: 1.921; 95% CIs:1.085–3.402). Many studies showed that micro-calcification was independently associated with malignancy^7,9,10^. However, there were fewer studies on macro-calcification in TNs. After 6.8 years’ observation of 480 asymptomatic papillary micro-carcinomas, Fukuoka *et al*. found macro-calcification significantly correlated with non-progressive disease^11^. In the current study, macro-calcification in TNs was a potential protective factor to some extent, while none macro-calcification was related with malignancy. Consolidation of macro-calcification may serve as a barrier against the carcinoma. Older patient age was shown as a protective factor in this study (*OR*: 0.982; 95%CIs: 0.967–0.998), which was consistent with previous studies^12,13^. Kwong *et al*.^12^ found that with advancing age, the prevalence of TNs increased, while the risk of malignancy decreased. Malignant nodules in patients age ≤45 yrs were twice as frequent as those >45 yrs in Bessey *et al*.’s study^13^.

Hammad *et al*.^14^ reported that nodules measured 30–59 mm in diameter had the greatest malignancy risk compared to those measuring <30 mm or >60 mm. Cordes *et al*.^15^ revealed nodule volume ≤2 ml was statistically significant for follicular neoplasms. Trimboli *et al*.^16^ reported that nodules >4 cm was an independent risk factor for malignancy with an OR of 2.1. By contrast, Unsal *et al*.^17^ thought nodule size ≥2 cm was not distinctive for diagnosis of malignancy. In the present study, nodule was smaller in malignant group than in benign group. When divided them into two groups by 20 mm, there was no statistically significantly difference in malignancy rate, which was similar to Unsal’s result^17^. Therefore, nodule size was excluded from independent risk factors of intermediate-risk TNs in binary multivariate logistic regression analysis in our study. The difference for the risk of nodule size might be attributed to the fact that the research object was limited to indeterminate risk TNs in the present study.

Intranodular vascularity, component, patient gender and nodule location did not achieve significant differences between benign TNs and malignant TNs in this study. It was reported that most thyroid cancers detected by US lacked intra-nodular vascularity^18^. Papillary thyroid carcinoma, accounting for most of the thyroid carcinomas, was not so invasive, which may explain its lack of vascularity. Batawil *et al*. recorded that solid structure could be predictive of malignancy^8^. No gender and location differences were found between benign and malignant TNs, which was in accordance with many previous studies^7,8,19–21^.

A final logistic regression predictive equation was developed in the present study. The results revealed that malignancy was depended on the US features such as indeterminate hyper-echoic spot, slight hypo-echogenicity, slightly ill-defined margin and none macro-calcification. The diagnostic performance of the equation, expressed as AUC, was statistically higher than any risk feature alone. In addition, a risk model with four stages (Stage I, Stage II Stage III to Stage IV) was established according to the four independent risk factors, and the corresponding risks of malignancy were 5.7%, 11.0%, 23.1%, 33.3% respectively. Our results indicated that from Stage I to Stage IV nodules, malignancy was gradually increasing. From Stage I to Stage II nodules, malignancy was relatively low, and follow-up was recommended. For Stage III and Stage IV nodules, we would recommend FNA. It’s believed that the risk mode could be potentially useful in clinical management of intermediate risk TNs.

There were still some limitations in our study. Firstly, selection bias may exist because patients included in the present study were scheduled for surgery or FNA. That means, this population is not representative of a whole population, the malignancy rate may be higher for the selection bias. Next, our study merely reflected single center’s experience. As a result, a multicenter study from different institutions and regions, particularly those with various thyroid cancer risks, is expected in the future. Thirdly, since it is a retrospective selection study, the statistical strength may be reduced, and a prospective study in the future is necessary to verify our findings. In addition, a follow-up of at least 6 months for benign FNA results was selected to exclude malignancy. Although this criterion was widely applied in many previous studies, many malignant lesions of the thyroid do not reveal an increase in size during that period. With the chosen time interval a benign nature seems probable but not proven. Moreover, the AUC of the prediction equation was not high enough, so that its diagnosis value was limited to some extent. More US features, such as US contrast-enhanced parameters and elastography parameters should be taken into account in further studies. Finally, it should be pointed out that thyroid malignancy especially PTCs may show ultrasound characteristics that are not in accordance with the specified risk factors.

## Conclusion

Among the intermediate-risk TNs of AACE/ACE/AME guidelines, special attention should be paid to the TNs with indeterminate hyper-echoic spot, slightly ill margin, slight hyper-echogenicity, or no macro-calcification. The probability of malignancy increased with the number of risk factors increasing. The proposed predictive model was potentially helpful in the clinic practice for the management of intermediate-risk TNs according to AACE/ACE/AME guidelines.

## Methods

### Patients

This retrospective study was approved by the Ethics Committee of the university hospital. Informed consent was waived for its retrospective nature. All procedures in this study were in strict compliance with the Declaration of Helsinki^22^.

From August 2015 to August 2016, 1224 consecutive patients with TNs were retrospectively enrolled. All the patients had US examinations. The patients were referred to US examination because of the following reasons: TNs discovered by palpation; follow-up of TNs; discomfort in the cervical region; TNs found incidentally in clinic. The inclusion criteria were as follows: (a) isoechoic or slightly hypoechoic; (b) round or ovoid, but without taller-than-wide shape; (c) well or slightly ill-defined, but without micro-lobulated or spiculated margins; (d) solid or predominantly solid nodules (i.e. cystic portion <50%); (e) diameter of calcification >1.0 mm^4^ if there was calcification, with or without acoustic shadow; (f) with or without hyperechoic spots of uncertain significance; (g) without extra-thyroidal growth; (h) patients underwent FNA or surgery after US examinations; (i) serum triiodothyronine (T_3_), thyroxine (T_4_), and thyroid stimulating hormone (TSH) in normal range. The exclusion criteria included: (a) incomplete image data or poor image quality (n = 99); (b) without follow-up or less than 6 months’ follow-up for those with benign cytological results (n = 114); (c) inadequate sampling of FNA (n = 21).

In general, only one nodule was selected for each patient and for those with multiple intermediate-risk TNs the largest one was selected. Finally, 1000 patients (222 males and 778 females, aged from 10–85 years, mean age: 52 years ± 13) with 1000 nodules (902 benign nodules and 98 malignant nodules, sized from 3–89 mm, median size: 16 mm) were included (Fig. 3).

### US examination and image analysis

US scanning was performed with Philips IU22 (5–12 MHz linear probe; Philips Medical Systems, Bothell, WA, USA), Siemens S2000 (5–14 MHz linear probe; Siemens Medical Solutions, Mountain View, CA, USA) or Logiq E9 (6–15 MHz linear probe; GE Medical Systems, Milwaukee, WI, USA) (Table 3) instruments by three radiologists who were board certified in thyroid US examination. All the US examinations were strictly complied with the same thyroid scanning protocol^20^. Firstly, patients were lying in supine gesture with complete exposure of their naked neck. The gain, frequency, focus position and depth were adjusted appropriately to make sure that the nodules were displayed clearly on the screen. Secondly, the target nodule and its surrounding thyroid tissue were scanned transversely and longitudinally. The US images of the nodule maximum diameter, margin, location, shape, internal echogenicity, component, echo uniformity, calcification, and vascularity were stored in the internal hard disk of the US instrument for subsequent analysis.

**Table 3:** The machine settings for the three different US scanners.

	Philips IU 22	Siemens S2000	Logiq E9
Transducer frequency	5–12 MHz linear	5–14 MHz linear	6–15 MHz linear
Gray scale			
Gain	1–100%	−20–20 dB	0–90 dB
Focus	33–45 Hz	10 fps-12 fps	10–12fps
Depth	2.5–8.0 cm	1–8 cm	2–15 cm
Dynamic range	46–76	30–90	30–120
Tissue harmonic mode	4.6–9.2 MHz	7.0,11.0,14.0 MHz	10,11,12,15 Hz
Color Doppler US			
Gain	1–100%	−20–19 dB	−20–30 dB
PRF	250–9000 Hz	100–9766 Hz	200–13100 Hz

US images were reviewed by another two radiologists with consensus. Patients’ general information, such as gender and age, were recorded. The US characteristics were evaluated as follows (Table 1): maximum diameter (>20 mm/≤20 mm); margin (well defined/slightly ill-defined); echogenicity (iso-echogenicity/slight hypo-echogenicity); location (left/right/isthmus); component (“predominantly solid” if more than 50% was solid/“solid” if it was entirely solid); echo uniformity (uniform/non-uniform); macro-calcification (present/absent); vascularity (Type I, no blood flow; Type II, predominantly peri-nodular blood flow; Type III, marked intra-nodular blood flow^23^); the indeterminate hyper-echoic spot inside the nodule (present/absent).

### Reference standard

All TNs were finally confirmed by either FNA biopsy or surgery. Pathological results after surgery were considered as the unique standard for malignant nodules. Benign lesions were confirmed by FNA and follow-up for at least 6 months without change in size and US features or pathological results after surgery.

US-guided FNA was performed under local anesthesia with a 22-gauge PTC needle (Hakko, Japan). About three to five pieces of smears were collected from each target nodule, which were kept in 95% alcohol and then submitted for haematoxylin-eosin staining. All reports were diagnosed by one of three experienced cytopathologists. The cytology was reported according to the Bethesda system for reporting thyroid cytopathologic findings^24^. The proportion of inadequate samplings was about 5% in our institution. Those nodules were recommended to undergo repeated FNA or diagnostic surgery depending on the suspicious features on US.

### Statistical analysis

Data were analyzed using the SPSS software (IBM Inc., Armonk,NY, USA; version 22.0) and MedCalc software (Mariakerke, Belgium; version 15.6). A two tailed *P* value <0.05 indicated statistically significant difference. Normal distributive continuous data were expressed as mean ± standard deviation (SD), while abnormal distributive continuous data were expressed as median (range interquartile). Categorical data were presented with counts (percentage). Normal distributive continuous data were compared by independent-samples *T* test, while abnormal distributive continuous data were compared by nonparametric independent-samples Mann–Whitney *U* test. Chi-square test or Fisher’s exact test was used to analyze the categorical variables.

Binary logistic regression analysis was performed to explore the risk factors for malignancy. Confidence intervals (CIs) were recorded as two-sided exact binomial 95% CIs. A logic regression predictive equation was obtained from the results. Receiver operating characteristic (ROC) curve analysis was used to evaluate the specificity and sensitivity. The best cut-off value for the predictive equation was achieved when Youden index (YI = sensitivity + specificity − 1) was the maximum. The diagnostic performances, expressed as area under ROC curve (AUC), for the statistically significant factors and the predictive equation were compared by MedCalc software.
